# Diphtheria and Filth

**Published:** 1876-04

**Authors:** 


### Selections.

### DIPHTHERIA AND FILTH.

There seems to be a growing impression that many of our epidemic diseases may originate in *filth*, a term which is applicable not merely to foul organic matter in solid masses, but also to minute portions of it suspended in water or in the air. One or more of these conditions appears to call out typhoid fever or diarrhœal diseases, and this relation has been so marked in many cases that filth has come to be regarded by some as the actual cause of their occurrence.

Whether or not this is true, and whether or not we have found the specific poison in such instances, does not appear to have so much practical importance, as the fact that we know the chief agencies by which infection is produced, and so may plan means to counteract them.

Acting upon the basis of these views, we should expect that approved sanitary measures would greatly mitigate the force of these diseases, and, in fact, this has really been accomplished, as shown by the statistical returns from villages and towns where sanitary regulations have been vigorously enforced.

The English reports are particularly valuable for the illustrations they afford of the working of these sanitary systems, especially in reference to typhoid fever and diarrhœal diseases. But while the attention of sanitary men has been so closely engaged in this especial direction, it can hardly be supposed that the element of filth may not also influence the occurrence of other affections. Indeed, these same reports tell us that outbreaks of both diphtheria and scarlet fever were closely associated with filth-nuisances of some sort or other. The evidence, however, is not altogether complete, for the instances of this kind are comparatively few, and, indeed, they are so far adverse to the filth theory, that the introduction of sanitary reforms was not followed by any diminution in the mortality from these sources.

It is, however, to be said, that statistics of diphtheria are liable to peculiar sources of error. In the first place, the name itself is a comparatively new one, not having appeared on the English registers before 1856, while in this city the occurrence of the first case is well remembered by some of our older physicians, though at the same time we have no good reason to suppose that the disease is a new one. Then, again, there has always been a division of opinion among the best practitioners as to the precise elements in a differential diagnosis of diphtheria, which, it is well known, in the milder forms presents uncommon difficulties. These facts will serve to explain why too much reliance should not be placed upon statistics gathered from a wide range of inquiry. The careful investigation of an outbreak in a single town, where the cases have been known to an intelligent physician, are of much more value. In this connection our readers will have noted that the last number of this journal contained an article bearing certain considerations on the origin of diphtheria. The writer has made an impartial selection of twenty houses where the disease had appeared in a single town, and he has directed his inquiries toward the condition of the cellars and underlying soil. All of the inspected houses were built on an alluvial formation, the cellars were generally damp or wet, and in all cases insufficiently ventilated. The greatest fatality occurred in those that were built upon filled land, where the subsoil was clay and the cellars were wet. In such cases the exhalations of the ground were naturally laden with the products of decomposition, and being undisturbed by currents of air, and in the presence of a moist atmosphere and mild temperature, were, in the writer's opinion, "accessory to, if not productive of, zymotic poisoning." The direction which these investigations took is interesting, and they point to results of practical importance.

In this city diphtheria seems to have been greatly influenced by conditions of a somewhat similar nature. In a large number of cases the possibility of infection from person to person has been almost inconceivable, especially when the poison seems to have such a limited capacity for transportation. Careful inspection of the premises in such cases has shown that sewer-gas could enter the apartments, either through defective drains, leaky pipes, or deficient traps; perhaps there was a lack of water to flush the basins or sinks, or foul matter had been allowed to collect on the premises or in the immediate neighborhood. These, or other unsanitary conditions, have almost invariably been found to be in operation when isolated cases have been investigated. Sometimes the filth nuisances were so plainly apparent that even non-professional persons believed them to have generated the sickness. These facts, of course, may not militate against the view that the poison has also a capacicty for conveyance. of which many excellent examples are given. And yet, if the filth origin of diphtheria be admissible, it is then easy to conceive how many persons in one house may take the disease, not from each other, but from the same foul source.

But while we are able so often to associate gross sanitary neglects with the origin of diphtheria, we must be alive to the fact that a hardly less censurable condition of things is often present in houses where we should least suspect it. Filth is not merely the companion of the poor and uncivilized. Indeed, so far from this being the case, many of the poorest families in the city observe rigid cleanliness in their homes, though the buildings they live in be pest-houses. The fault may be with their landlord, who, perhaps, has given them leaky pipes, a scanty supply of water for their basins or sinks, improper water-closets, or has allowed the cellars or yards to be receptacles for all manner of foulness. Nor is it these landlords only, but the masters of substantial and elegant residences in the better portions of our city, who, either from indifference or ignorance of the commonest sanitary rules, leave the doors wide open for the entrance of disease. Privies that are used by their subordinates are allowed to get out of repair, and are then heedlessly used until the nuisance becomes so excessive that the evil betrays itself.

Ashes and garbage also are sometimes allowed to collect in the areas or basements, until it suits the convenience of the servants or masters to have them removed. Nor is it very uncommon for the better class of houses to have worn out soil-pipes, which breathe out pestilence whenever there is pressure of sewer-gas. The common lead soil-pipes, with which so many of our houses are fitted, is now known to last but a comparatively short time. Under the influence of sewer-gas alone there is a gradual corrosion of the lead, and this action appears to be rapid or slow according to the amount of pressure. When such pipes are removed they are often so thin that a pin can be pushed through them, and even have large holes at the bends. These pipes are often hid away in the walls, and their condition is not known until a serious break has taken place, but meantime they have been pouring out their noisome vapors into the house. Nor is this all, for there are those living among us in good circumstances who altogether abuse the advantage which a good water system has given us. Such persons would scorn to be accused of anything uncleanly, but at the same time will sometimes not hesitate to use water-closets when out of order, and often fail to properly flush them sufficiently to carry off the waste beyond the trap, even when the water supply is abundant. When such water-closets are in the vicinity of sleeping rooms, and have imperfect communication with the outside air, the evil becomes an intolerable nuisance.

It is these dangers, together with the difficulty of at all times keeping back the sewer-gas from houses, that has led to a common notion that water closets and wash sinks and basins are so detrimental in their operation that the safest plan is to remove them as far as possible from dwelling houses, or at least to reduce them to a minimum, and return to the old pitcher-and-basin system. This, however, appears to be carrying the matter to extremes. Authorities appear to be agreed that for our cities, at any rate, the water system offers the best advantages, and is the most effective way of removing waste.

Another possible source of danger is our drinking water. As it comes through our direct pipes it is pretty sure to be sound and healthy, both because it comes to us from a comparatively pure source and it is robbed of its organic particles during its passage down. But we are apt to forget that in our own houses the water may be contaminated in various ways. One of the most common is for the overflow pipe from the tank to communicate with the sewer pipe, so that thus the sewer gas is led directly to the water supply.

A soil drenched with organic matter is also capable of giving out noxious gases, as pointed out by our recent contributor, and it is in such neighborhoods that diphtheria has shown a tendency to prevail—parts where there is neither sewerage nor drainage, and where the waste can collect in pockets on the surface or soak away into a spongy soil.

Many persons do not appear to be aware that these sanitary abuses are constantly in existence about us, but they are matters to which public attention should be turned, that they may be the sooner abated.—*The Medical Record.*

